# Exploitation of the Toll-like receptor system in cancer: a doubled-edged sword?

**DOI:** 10.1038/sj.bjc.6603275

**Published:** 2006-08-01

**Authors:** S D Killeen, J H Wang, E J Andrews, H P Redmond

**Affiliations:** 1 Department of Academic Surgery, National University of Ireland (NUI)/University College Cork (UCC), Cork University Hospital, Cork, Ireland

**Keywords:** toll-like receptors, toll-like receptor ligands, NF-*κ*B, innate immunity, immunotherapy

## Abstract

The toll-like receptor (TLR) system constitutes a pylogenetically ancient, evolutionary conserved, archetypal pattern recognition system, which underpins pathogen recognition by and activation of the immune system. Toll-like receptor agonists have long been used as immunoadjuvants in anti cancer immunotherapy. However, TLRs are increasingly implicated in human disease pathogenesis and an expanding body of both clinical and experimental evidence suggests that the neoplastic process may subvert TLR signalling pathways to advance cancer progression. Recent discoveries in the TLR system open a multitude of potential therapeutic avenues. Extrapolation of such TLR system manipulations to a clinical oncological setting demands care to prevent potentially deleterious activation of TLR-mediated survival pathways. Thus, the TLR system is a double-edge sword, which needs to be carefully wielded in the setting of neoplastic disease.

## METHODS

Referenced papers were identified using multiple electronic searches of the Medline database with the keywords (alone or in combination): toll-like receptors (TLRs), cancer, TLR ligands, NF-*κ*B, innate immunity, acquired immunity and immunotherapy. The search strategy incorporated MESH terms, and results were evaluated for sensitivity and specificity. Additional articles were identified from the references of retrieved papers. Papers were included on the basis of evidence supporting individual points.

## BACKGROUND

A total of 13 mammalian toll paralogues (11 of which are expressed in humans) termed TLR have been described (O'Neill, 2006). Toll-like receptors expressed on immune system sentinel cells such as dendritic cells and macrophages sense a range of chemicals produced by bacteria, viruses, fungi and protozoa. For example lipid-based predominantly bacterial structures such as mycobacterial lipopeptides and lipopolysaccaride are recognised by TLR2 (in combination with TLR1 or TLR6 as heterodimers) and TLR4 (as a homodimer), respectively, while viral and/or bacterial nucleic acids are recognised by TLR3 (stranded RNA (dsRNA)), TLR7, TLR8 and TLR9 (CpG motifs in DNA). Toll-like receptors are also extensively expressed on other cell types and may have endogenous ligands (Cook *et al*, 2004).

Subsequent TLR-mediated intracellular signalling broadly involves two transduction pathways, which ultimately facilitate activation of the transcription factor NF-*κ*B, a master switch for inflammation, regulating the transcription of genes encoding proteins involved in immunity and inflammation or the MAP kinases, p38 and Jun amino-terminal kinase (JNK), which participate in increased transcription and also manipulate the stability of mRNAs that contain AU repeats (McGettrick and O'Neill, 2004).

All TLRs activate NF-*κ*B and MAP kinases via a pathway that involves IRAK-4 and IRAK-1. *Fundamental to NF-κB translocation is IKK kinase* (*composed of catalytic subunits IKK1 and IKK2 and regulatory subunit NEMO* (*NF-κB essential modulator*)) *activation. IKK phosporylates the NF-κB inhibitory dimer* (*consisting of various p65, p50 subunit combinations*). This process is controlled by the differential recruitment of four adaptor proteins namely MYD-88, Mal (MyD88 adaptor-like), TRIF (TIR-related adaptor protein inducing interferon) and TRAM (Trif-related adaptor molecule) to TLRs. This selective adaptor protein recruitment is responsible for the different gene expression profiles evident when individual TLRs are stimulated (O'Neill, 2006).

## TLRS AND HUMAN DISEASE PROCESSES

Toll-like receptors are increasingly implicated in disease pathogenesis (Cook *et al*, 2004). Population studies of polymorphisms in genes encoding TLRs or their downstream signalling molecules in conjunction with limited extrapolation from animal studies have directly linked these pathways to multiple human disease processes.

As intuitively expected, many of these polymorphisms are implicated in infection and sepsis. The initially identified and best studied D299G polymorphism (an amino acid substitution, from aspartic acid to glycine at position 299 in the TLR-4 gene) is associated with an increased frequency of Gram-negative infection and increased severity of Gram-negative sepsis (Child *et al*, 2003). Meningococal infections in an ethnically matched population have also been linked to a rare, heterozygous missense TLR4 mutation (Smirnova *et al*, 2003). The R753Q polymorphism in TLR2 is linked with reduced response to peptides from *Borrelia burgdorferi* and *Treponema pallidum*, and may predispose to *Staphylococcal* infection or tuberculosis (Ogus *et al*, 2004) while the R677W polymorphism in TLR2 which impairs activation of NF- B by *Mycobacterium leprae* and *Mycobacterium tuberculosis* apparently enhances susceptibility to leprosy and tuberculosis (Cook *et al*, 2004).

Conversely the TLR-4 D299G polymorphism is associated with decreased risk of carotid artery atherosclerotic stenosis and acute coronary events (Kiechl *et al*, 2002).

TLR polymorphisms may underline aspects of asthma and certain autoimmune conditions. Although the D299G polymorphism has no effect on the overall incidence of asthma (Raby *et al*, 2002), for asthma specifically associated with house dust mite LPS, people with the D299G polymorphism had a decreased risk of bronchoreactivity (Werner *et al*, 2003) while asthmatic people with this polymorphism have an increased severity of atopy (Yang *et al*, 2004).

TLR signalling abnormalities have also been implicated in immune-deficency conditions. Mutations in gene encoding NEMO (primarily in exon 10) lead to anhidrotic extodermal dysplasia (Puel *et al*, 2004) and a missense mutation involving serine 32 of I B additionally produces anhidrotic extodermal dysplasia with immunodeficiency (Courtois *et al*, 2003).

Overall the expanding body of data has demonstrated important relationships between TLR signalling pathways and human disease. Further advances in genetic profiling, intracellular transduction pathways and effector mechanisms may permit focused targeting of specific elements of the TLR signalling in theses disease states.

## TLRS AND ANTICANCER IMMUNOTHERAPY

Immunotherapy is an evolving fourth prong to the anticancer armamentarium and represents a potentially all pervading targeted nontoxic mechanism to eradicate even micrometastatic disease (Mocellin *et al*, 2004). Regrettably harnessing the undisputed potential of the immune system has proven problematic (Gilboa, 2004).

Extensive research has focused on anticancer vaccination whereby a tolerant immune system is primed to recognise and destroy tumour cells. Fundamental to potentiation of immune responses to tumour-associated antigen (Ag) is the use of adjuvants, which foster efficient antibody (Ab) production and killer cell induction (Cheadle and Jackson, 2002).

Since the pioneering work of William Coley, with his eponymously named Coley's toxin, attempts to exploit the immune system using immunopotentiators abound, with microbial products extensively utilised for this purpose (Coley, 1893).

Until recently the underlying mechanism of action of many immunoadjuvants has remained undefined, possibly impairing optimal utilisation. Increased understanding of TLR signalling has allowed elucidation of the molecular basis of such adjuvant activity with the potential for therapeutic application.

**Figure 1:**
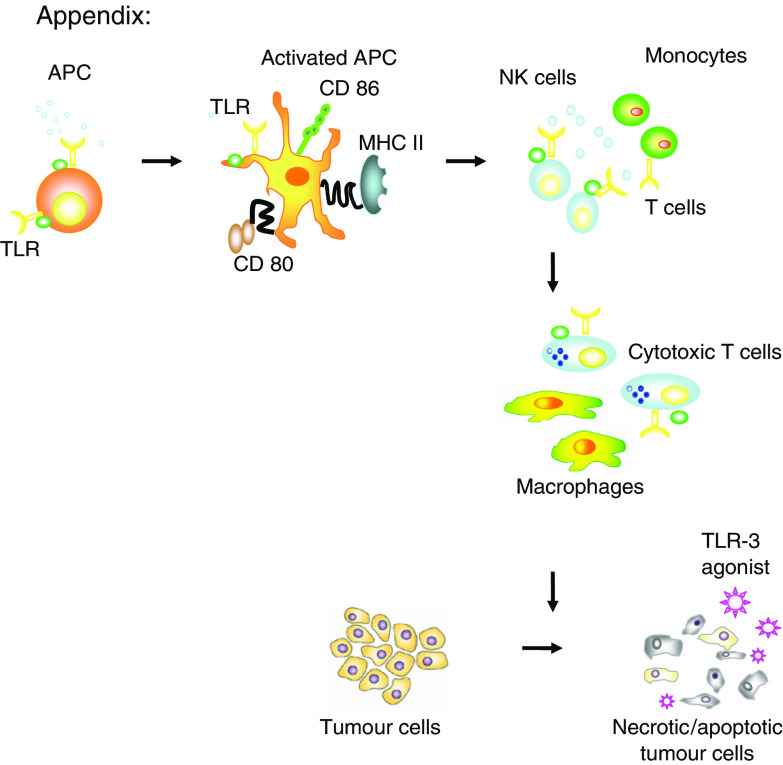
TLR agonists can activate APC, macrophages and cytotoxic T cells potentially leading to tumour destruction. TLR agonists can also directly promote tumour cell apoptosis. (TLR-4;Toll-like receptor 4, APC; antigen presenting cells, MHC; major histocompatability complex).

Thus, it is now apparent that the maturation of dendritic cells (DCs) and cytokine induction underpinning the antineoplastic activity of *Bacillus Calmette-Guerin cel wall skeleton (BCG-CWS)* is mediated by both TLR2 and TLR4 (Tsuji *et al*, 2000) while the biological response modifier OK-342 is dependent on the presence of functioning TLR-4 for its anticancer effect (Okamoto *et al*, 2003). Double-stranded RNA (dsRNA) activates TLR3 on DCs to release type 1 IFN that induces tumour cell apoptosis and NK-mediated tumour cytotoxicity (Whitmore *et al*, 2004) (Figure 1). Oligodeoxynucleotides (ODN) with unmethylated deoxycytidyl-deoxyguanosine (CpG) dinucleotides (CpG ODN) mimic the immunostimulatory activity of bacterial DNA recognised by the Toll-like receptor 9 (TLR9) and are potent adjuvants utilised in cancer therapy (Jahrsdorfer *et al*, 2005). *Imiquimod* is a TLR-7 agonist extensively used in the treatment of basal cell carcinoma (Urosevic *et al*, 2005).

Recent studies suggest that adjuvants may evoke different immune responses by targeting distinct TLRs and their intracellular adaptors. Toll-like receptors appear to segregate into functional subgroups, based on their abilities to modulate particular types of innate and adaptive immune responses. Toll-like receptor-7, -8 and -9 induce IFN-*α*, promote efficient cross-presentation, cytotoxic T-lymphocyte activation and polarised Th1 responses (Hopkins and Sriskandan, 2005). Signalling via TLRs-3 and -5 also yield a Th1 response and weaker cytotoxic T-cell reaction while signalling through TLR-4 generates a response with an atypical Th1 bias (Agrawal *et al*, 2003). In contrast, many TLR-2 ligands appear to show a Th2 bias (Agrawal *et al*, 2003). Certain immune responses can prove beneficial, while others, deleterious in the neoplastic setting. Thus, it may be possible to engineer the most appropriate, antitumour immune response by selecting certain TLR ligands individually, as a cocktail or in conjunction with other modalities (Koski and Czerniecki, 2005). Moreover, in addition to selecting a desired immune response it may also be feasible to break the stranglehold of clone specific immunosuppressive regulatory T-cells (T-regs) extensively implicated in deleterious immune suppression in through explicit targeting of TLR-8 (Peng *et al*, 2005).

Furthermore recent studies demonstrated that certain tlr agonists can directly promote tumour cell death. Salaun *et al* showed that synthetic (double stranded) dsRNA induces apoptosis of human breast cancer cells in a TLR3-dependent manner, which involves the molecular adaptor Toll/IL-1R domain-containing adapter inducing IFN-beta and type I IFN autocrine signaling (Salaun *et al*), (Figure 1).

## TLRS AND CANCER PATHOGENESIS

Analogous to other disease processes direct clinical data indicating a role for TLRs in cancer progression is scant. Zheng *et al* (2004) demonstrated a significant association between prostate cancer and a TLR-4 sequence variant (11381G/C). The variant frequency was markedly higher in prostate cancer patients compared to controls and was higher again in early onset cases. Compared to men with the wild-type genotype, the sequence variant conferred an overall increased risk of 26% for prostate cancer. This group also demonstrated a statistically significant relationship between high risk TLR-4, 6 and 10 haplotype-tagging SNPs (htSNPs) and prostate cancer in the same population suggestive of a founder prostate cancer risk variant on this haplotype background (Sun *et al*, 2005).

An escalating mass of experimental evidence suggests that the neoplastic process may sabotage TLR signalling pathways to advance cancer progression. Contemporary *in vivo* genetic studies highlight a more fundamental and direct role for epithelial cell TLRs and NF-*κ*B in tumour development. Multiple chronic inflammatory diseases have been associated with cancer development including inflammatory bowel disease, hepatitis and chronic *Helicobacter pylori* infection predisposing to colorectal, hepatocellular and gastric cancer respectively. The published literature overwhelmingly supports a scenario in which inflammation promotes (rather than initiates) carcinogenesis in a noncell-autonomous fashion (Coussens and Werb, 2002). Current doctrine holds that at sites of inflammation, cytokines, chemokines and matrix degrading enzymes are released in a persistent unregulated manner predominantly by cells of the innate immune system serving to enhance angiogenesis, invasiveness and promote survival of pre-malignant and neoplastic cells. However, Rakoff-Nahoum *et al* (2004) demonstrated that TLR-2, TLR-4 and MyD88.deficient mice had an unexpectedly increased susceptibility to *dextran sulfate sodium* (DSS) colon injury. In this model commensal bacteria activate TLRs, and the resulting TLR activity provides protection from colitis-induced damage. Recognition of bacterial products occurs by TLRs expressed on enterocytes, residing in an intact epithelial sheet and such activated TLRs then mediate a cell-autonomous NF-κB dependent cell survival response. Greten *et al* (2004) chemically induced colon carcinomas in mice that do not express IKK2 in their intestinal epithelial cells. They showed that IKK2-knockout (KO) in intestinal epithelial cells led to a decrease in tumour incidence (but not tumour size) which was associated with enhanced epithelial cell apoptosis. Conversely when IKK2 was specifically deleted in myeloid cells, the chemically induced colon carcinomas were reduced in size leading the authors to conclude that, in enterocytes, IKK2 contribute to tumour promotion by suppressing apoptosis, whereas IKK2 KO in myeloid cells leads to downregulation of proinflammatory, tumour-promoting mediators.

**Figure 2:**
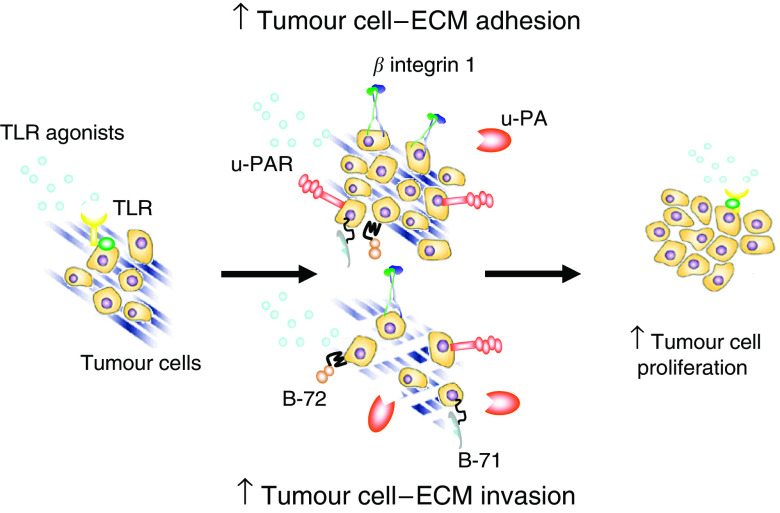
TLR agonists can directly promote enhanced tumour cell adhesion and invasion through the upregulation of proteases such as the urokinase plasminogen activator system and beta integrin 1, ultimately facilitating tumour progression (TLR-4;Toll-like receptor 4, u-PA: urokinase plasminogen activator, pro-u-PA: pro-urokinase plasminogen activator (zymogen), u-PAR; urokinase plasminogen activator receptor, MMP; matrix metalloprotease, pro-MMP, promatrix metalloprotease (zymogen)).

Contemporary studies have established a decisive role for bacterial endotoxin or lipopolysaccaride (LPS) the prototypical TLR-4 ligand in the increasingly recognised phenomenon of surgery induced accelerated tumour growth. Following laparotomy and air laparoscopy, LPS contaminates the peritoneal cavity and enters the systemic circulation due to perioperative bacterial gut translocation. In a murine metastatic breast carcinoma model, mice undergoing laparotomy or air laparoscopy had increased serum levels of LPS and metastatic tumour burden compared to those in the CO2 laparoscopy group *(which was not contaminated with endotoxin)*, a result replicated by intraperitoneal LPS injection (Pidgeon *et al*, 1999; Harmey *et al*, 2002). Luo *et al* used TLR-4 deficient CT-26 murine colorectal cancer cells in their study and suggested that the tumour promoting effect of LPS is subservient to inflammatory cytokines produced primarily by the innate immune system, as the LPS-induced enhanced tumour burden was TNF-*α* dependent and abolished with super-repressor-mediated NF-*κ*B inhibition. Moreover the effect was abolished in TLR-4 deficient mice highlighting the importance of TLRs as necessary initiators of this phenomenon (Luo *et al*, 2004). While this association between LPS and accelerated perioperative tumour growth may be a manifestation circuitous immunologically mediated singularity, a direct TLR-4 dependent effect on cancer cells may also be involved. Huang *et al* have shown the presence of TLRs on most cancer cell types (Huang *et al*, 2005). Andrews *et al* (2001) and Wang *et al* (2003) demonstrated that LPS may also promote tumour progression by acting directly on cancer cells resulting in an NF-*κ*B mediated *beta 1 integrin* dependent increased *in vitro* tumour cell endothelial cell adhesion, tumour cell extracellular matrix adhesion and tumour cell extracellular matrix invasion. (Figure 2). *H*. *pylori* acting via TLR2/TLR9 on gastric epithelial cells activated both the PI-PLCgamma/PKCalpha/c-Src/IKKalpha/beta and the NIK/IKKalpha/beta cascade resulting in increased expression of COX-2 which could potentially contribute to the gastric cancer progression (Chang *et al*, 2004).

Furthermore the TLR-signalling pathway may be manipulated by tumour cells in a manner analogous to the mouse mammary tumour retro virus which subverts the innate immune system through the TLR-4 dependent production of the immune suppressive cytokine IL-10 (Jude *et al*, 2003). del Fresno *et al* (2005) using an *in vitro* system, indicated that tumour cells deactivate human monocytes through the release of hyaluronan which upregulated monocyte IRAK-m in a CD 44 and TLR-4 dependent manner. This tumour-mediated exploitation of TLR signalling may also apply to the adaptive immune system. Huang *et al* established that activation of TLR-4 signalling in tumour cells by lipopolysaccharide induced the synthesis of various soluble factors and proteins including interleukin-6, inducible nitric oxide synthase, interleukin-12, B7-H1, and B7-H2 resulting in resistance of tumour cells to cytotoxic T-lymphocyte (CTL) attack. Blockade of the TLR-4 pathway reversed tumour-mediated suppression of T-cell proliferation and natural killer cell activity *in vitro*, and *in vivo*, delaying tumour growth and prolonging survival of tumour-bearing mice (Huang *et al*, 2005). Taken together such findings indicate that tumour cell TLR signalling may be subverted by the neoplastic process resulting in a cascade leading to enhanced tumour cell invasion and immune surveillance evasion.

**Figure 3:**
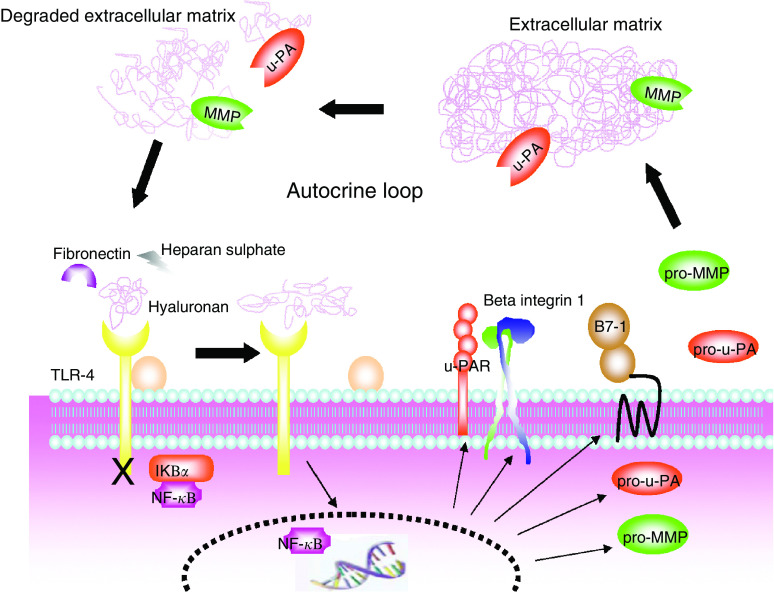
A putative self perpetuating, neoplastic paracrine loop, whereby protease degraded extracellular matrix components (ECM) release the tumour cell TLR signalling apparatus from inhibition and stimulate this pathway, culminating in enhanced protease production and hence ECM breakdown. *(TLR-4;Toll-like receptor 4, u-PA: urokinase plasminogen activator, pro-u-PA: pro-urokinase plasminogen activator (zymogen), u-PAR; urokinase plasminogen activator receptor, MMP; matrix metalloprotease, pro-MMP, promatrix metalloprotease (zymogen)).*

Allied to this is a reputed role for TLRs in the immune response to self-molecules that have in some way been altered from their native state or accumulated in nonphysiologic sites or quantities. Several putative endogenous ligands have been reported including hsp60, hsp70, gp96, fibrinogen, surfactant protein A, the extra domain A of fibronectin, HMGB-1, Heparan sulphate, hyaluronan and B_2_-defensin (Rifkin *et al*, 2005). Studies suggest a role for such compounds, in pathologies as diverse as pancreatitis and haemorrhage-induced lung injury (Johnson *et al*, 2004). A provocative hypothesis proposed by Zeh *et al* states that cancer is a disorder of cell death rather than cell growth propelled by the release of necrotic products such as HMGB-1 to which tumour cells become ‘addicted’. Such products could possibly act through TLRs expressed by cancer cells. Therefore, other endogenous ligands which are increased in the tumour environment could also modify both the local inflammatory response at the tumour site and the rate of tumour-cell growth (Zeh and Lotze, 2005). Although ligands that drive TLR-mediated tumour cell immune suppression have yet to be identified, endogenous compounds could well be involved (Huang *et al*, 2005). Johnson *et al* have recently demonstrated that signalling through TLR4 function is apparently strongly inhibited by intact extracellular matrix (ECM) and this inhibition is abrogated and endogenous agonist(s) liberated when the matrix is degraded (Brunn *et al*, 2005). Thus, escape from ECM mediated inhibition, due to ECM breakdown caused by proteases released in the course of infection or injury, may be the critical first event by which TLRs initiate immune responses. By extrapolation this pathway *could also be* utilised by cancer cells to institute a self propagating autonomous autocrine loop. Thus, protease secretion by cancer cells leads not only to extracellular matrix degradation with enhanced tumour cell invasion but also release of tumour cell TLR apparatus from matrix constrains and liberation of putative TLR ligand. This culminates in TLR stimulation, corruption of the intracellular signalling pathways and the production of tumour promoting compounds including proteases. Such proteases promote tumour progression and further degrade the extracellular matrix perpetually maintaining this proposed autocrine loop ad infinitium (see Figure 3). Speculation regarding any possible association of endogenous TLR ligands in tumour progression must, however, be tempered by concerns regarding possible confounding contaminating microbial *pathogen associated molecular patterns* (PAMPs) such as LPS (Tsan and Gao, 2004; Geisler *et al*, 2005) and *also a putative direct cytotoxic effect of certain TLR agonists on cancer cells*. Collectively these studies suggest that *certain* PAMPs and endogenous compounds acting directly through TLRs on transformed cells can trigger the NF-*κ*B survival pathway serving to upregulate a plethora of antiapoptotic, proinvasive and immune evasive mechanisms. Subversion of the TLR signalling pathway by neoplasms constitutes a new paradigm in cancer progression, one open to potentially beneficial manipulation with careful intervention.

## FUTURE DIRECTIONS

Recent advances in the field of TLR signalling have opened a multitude of oncological therapeutic opportunities. Given the apparent appropriation of certain immune responses by TLR subgroups it may be possible to generate a desired immune response in cancer patients based on adjuvant TLR activity. The counterproductive or even deleterious stimulation of TLRs on tumour cells could be avoid by profiling the TLR status on tumour cells and selecting adjuvants which will be recognised by immune cell but not cancer cell TLRs thereby preventing activation of the prosurvival TLR signalling pathways in cancer cells. The subversion of the TLR system by the neoplastic process is another valid interventional target. However, targeting of TLRs may potentially disrupt beneficial host immunity and homeostatic functions, provoke damaging autoimmune phenomena and interfer with concurrent therapeutic regimens. Furthermore putative endogenous TLR ligands need to be identified, the precise details of ligand–receptor interactions and their contribution to cancer pathogenesis, clarified.

## CONCLUDING REMARKS

The TLR system is a keystone of immunoadjuvant therapy. With further understanding of TLR function, signalling and effector mechanisms, manipulation of this pathway holds great potential in cancer treatment. However, such strategies are not without risk. The TLR system of immune and cancer cells alike, is actively subverted by the neoplastic process to advance disease propogation and this system coupled with putative endogenous ligands may actually comprise a novel paracrine self propagating feedback loop. Hence, the TLR system constitutes a double edge sword that needs to be carefully wielded in the setting of neoplastic disease.
